# The role of neurotransmitters in mediating the relationship between brain alterations and depressive symptoms in patients with inflammatory bowel disease

**DOI:** 10.1002/hbm.26439

**Published:** 2023-08-02

**Authors:** Jun Wang, Guangyao Liu, Kun Xu, Kai Ai, Wenjing Huang, Jing Zhang

**Affiliations:** ^1^ Department of Magnetic Resonance Lanzhou University Second Hospital Lanzhou China; ^2^ Second Clinical School Lanzhou University Lanzhou China; ^3^ Gansu Province Clinical Research Center for Functional and Molecular Imaging Lanzhou University Second Hospital Lanzhou China; ^4^ Deparment of Clinical and Technical Support, Philips Healthcare Xi'an China

**Keywords:** Crohn's disease, functional MRI, inflammatory bowel disease, magnetic resonance spectroscopy, metabolite, resting state, ulcerative colitis

## Abstract

A growing body of evidence from neuroimaging studies suggests that inflammatory bowel disease (IBD) is associated with functional and structural alterations in the central nervous system and that it has a potential link to emotional symptoms, such as anxiety and depression. However, the neurochemical underpinnings of depression symptoms in IBD remain unclear. We hypothesized that changes in cortical gamma‐aminobutyric acid (GABA+) and glutamine (Glx) concentrations are related to cortical thickness and resting‐state functional connectivity in IBD as compared to healthy controls. To test this, we measured whole‐brain cortical thickness and functional connectivity within the medial prefrontal cortex (mPFC), as well as the concentrations of neurotransmitters in the same brain region. We used the edited magnetic resonance spectroscopy (MRS) with the MEGA‐PRESS sequence at a 3 T scanner to quantitate the neurotransmitter levels in the mPFC. Subjects with IBD (*N* = 37) and healthy control subjects (*N* = 32) were enrolled in the study. Compared with healthy controls, there were significantly decreased GABA+ and Glx concentrations in the mPFC of patients with IBD. The cortical thickness of patients with IBD was thin in two clusters that included the right medial orbitofrontal cortex and the right posterior cingulate cortex. A seed‐based functional connectivity analysis indicated that there was higher connectivity of the mPFC with the left precuneus cortex (PC) and the posterior cingulate cortex, and conversely, lower connectivity in the left frontal pole was observed. The functional connectivity between the mPFC and the left PC was negatively correlated with the IBD questionnaire score (*r* = −0.388, *p* = 0.018). GABA+ concentrations had a negative correlation with the Hamilton Depression Scale (HAMD) score (*r* = −0.497, *p* = 0.002). Glx concentration was negatively correlated with the HAMD score (*r* = −0.496, *p* = 0.002) and positively correlated with the Short‐Form McGill Pain Questionnaire score (*r* = 0.330, *p* = 0.046, uncorrected). There was a significant positive correlation between the ratio of Glx to GABA+ and the HAMD score (*r* = 0.428, *p* = 0.008). Mediation analysis revealed that GABA+ significantly mediated the main effect of the relationship between the structural and functional alterations and the severity of depression in patients with IBD. Our study provides initial evidence of neurochemistry that can be used to identify potential mechanisms underlying the modulatory effects of GABA+ on the development of depression in patients with IBD.

## Graphical abstract

GABA+ and Glx concentrations significantly decreased in the brain region of the media prefrontal cortex in patients with IBD. GABA+ mediates the main effect of the relationship between the structure/function alterations with the severity of depression in patients with IBD.

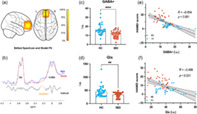

**Abbreviations**
^1^H‐MRS: proton magnetic resonance spectroscopy
BMI: body mass index
CD: Crohn's disease
FP: frontal pole
GABA+: gamma‐aminobutyric acid plus other neuroactive substances
Glx: glutamate + glutamine
GSRS: Gastrointestinal Symptom Rating Scale.
HAMA: Hamilton Anxiety Rating Scale
HAMD: Hamilton Depression Rating Scale
IDBQ: Inflammatory Bowel Disease Questionnaire for Quality of Life
MoCA: Montreal Cognitive Assessment
mPFC: medial prefrontal cortex
NA: not available
PC: precuneus cortex
PCC: posterior cingulate cortex
PCS: Peritraumatic Distress Inventory
PSQI: Pittsburgh Sleep Quality Index
rmOFC: right medial orbitofrontal cortex
rPCC: right posterior cingulate cortex
SF‐36: Short Form‐36 Health Survey
SF‐MPQ: Short‐Form McGill Pain Questionnaire
TIV: total intracranial volume
UC: ulcerative colitis
VAS: Visual Analog Scale
VSI: Visceral Sensitivity Index

## 1 INTRODUCTION

Inflammatory bowel disease (IBD) is a chronic intestinal inflammation disease characterized by recurrent episodes and remission of inflammation (Kluthe et al., 2018). It refers to a group of diseases that includes Crohn's disease (CD) and ulcerative colitis (UC) (Golusda et al., 2022; Moldovan et al., 2023). IBD affects millions of people worldwide and it is associated with a high burden of physical, psychological, and social symptoms (Loftus Jr., 2004). Depression, which is one of the most common comorbidities of IBD, is a difficult‐to‐treat neuropsychiatric disorder that is influenced and regulated by a variety of neurochemical mechanisms (Geissler et al., 1995; Goodyear et al., 2022; Morozova et al., 2022; Thomann et al., 2017; Thomann et al., 2022; Wang et al., 2022). Studies have reported that 21%–27% of patients with IBD suffer from depression, which can have a substantial impact on their overall health and disease management (Craig et al., 2022), and even increase the frequency of the active phase (Marrie et al., 2021). Various studies have reported inconsistent estimates of the co‐occurrence of depression or anxiety with IBD, and the causative mechanism and direction of this association remain uncertain (Barberio et al., 2021). The intricate connection between the gut and the brain, also known as the “gut–brain axis” or “gut‐microbiota–brain axis,” increases the likelihood of mental disorders in patients with IBD, compared with patients with other chronic diseases due to the function of afferent activation (Bisgaard et al., 2022). Animal studies indicate that induced colitis can trigger changes in the brain, resulting in behavioral abnormalities, such as anxiety, depression, and cognition. These changes may be caused by an impaired central NO pathway (Heydarpour et al., 2016), increased production of proinflammatory cytokines in the brain (Haj‐Mirzaian et al., 2017), elevated hippocampal neurodegeneration (Zonis et al., 2015), changes in cerebral blood vessels (Carloni et al., 2021), or a combination of these mechanisms. Depression can worsen the physical symptoms of IBD, increase the risk of hospitalization, and reduce the effectiveness of treatment (Gao et al., 2021; Taft et al., 2009). Current treatments for depression in patients with IBD, such as antidepressants and psychotherapy, are often limited in their effectiveness (Hu et al., 2021). Our understanding of the fundamental neuropathology of enteritis‐related emotional disorders has advanced significantly over the last 20 years (Guthrie et al., 2002), the neurochemical mechanisms that underlie the symptoms of depression in IBD remain poorly understood (Alexakis et al., 2017; Dolapcioglu & Dolapcioglu, 2015).

Studies in depressed patients and rodent models demonstrate that depression and chronic stress exposure cause atrophy of neurons in cortical and limbic brain regions implicated in depression, which is mainly located in hippocampus and the prefrontal cortex (PFC) (Evans et al., 2018; MacQueen et al., 2008). This series of changes can be captured through neuroimaging methods, including decreased volume and connectivity of hippocampus and PFC (Savitz & Drevets, 2009). Autopsy studies confirm reduced neuronal cell volume and decreased glial cell in the PFC of depressed patients (Rajkowska & Stockmeier, 2013). Functional imaging studies reveal heightened default mode network (DMN) activity and diminished salience network (SN) and central executive network (CEN) function in depressed individuals. This aligns with increased rumination and introspection, alongside reduced engagement with external stimuli (Greicius et al., 2007). Research has shown that the mPFC is involved in regulating various cognitive and emotional processes (Radley et al., 2009; Sun et al., 2019). Its imbalance of excitation and inhibition is related to severe depression and chronic stress (Page & Coutellier, 2019). Gamma‐aminobutyric acid (GABA, generally called GABA+) and glutamate (Glu; combined measures of glutamate and glutamine are usually called Glx) are major inhibitory and excitatory neurotransmitters that modulate neural activity and human behavior (Foerster et al., 2015; Strasser et al., 2020). The MEGA‐PRESS (Magnetization‐Encoded Gradient Adapted Spectroscopy‐PRESS) protocol allows non‐invasive, in vivo detection of GABA+ and Glx in the brain (Petroff et al., 2000; Schur et al., 2016). The concept of excitatory/inhibitory (E/I) balance in the brain can be simplified as the ratio of Glx to GABA+ (Rideaux, 2021). The changes in brain connectivity in patients with depression may be related to the imbalance of E/I, synaptic plasticity damage, and nerve transmission interruption, which have internal and external control over the information flow in the brain (Abdallah et al., 2014). Recent studies using in vivo proton magnetic resonance spectroscopy (MRS) have more consistently reported a decrease in glutamate metabolite levels in the medial frontal cortex in depression patients (Moriguchi et al., 2019). Studies combining MRS and fMRI have shown that a decrease in glutamate in the anterior cingulate cortex (ACC) is associated with a decrease in connectivity to the insula and a decrease in bold responses to emotional stimuli in major depressive disorder patients (Lener et al., 2017). Preclinical studies on chronic stress have also reported reduced levels of GABA synthase and neuropeptides in medial PFC and other cortical brain regions (Fee et al., 2017). This accumulating evidence highlights the significant role of the mPFC as a crucial brain region in the context of depression.

Extensive studies have indicated that patients with IBD may have structural and functional changes in key areas of the brain related to depression symptoms (Agostini et al., 2011; Agostini et al., 2013; Cisler, 2017; Meng et al., 2022; Page & Coutellier, 2019; Peppas et al., 2021; Thomann et al., 2021). Patients with UC who are in remission exhibit decreased activity in the amygdala, thalamus, and cerebellum regions in response to positive emotional stimulation, compared with a healthy control group (Agostini et al., 2011). According to a study of dynamic functional connectivity, the stability of the medial prefrontal cortex (mPFC) cortical region in patients with UC was found to be elevated, and there was a significant positive correlation with levels of depression and anxiety (Wang et al., 2022). Researchers conducting a study on patients with CD observed low functional connectivity in the bilateral hippocampal limbic system, which may suggest a decrease in the ability of the limbic system to regulate visceral sensations and pain in these patients (Bao et al., 2018). Thomann et al. used functional connectivity analysis to examine multiple instances of intrinsic neural network dysfunction in patients with CD in remission (Thomann et al., 2017). They detected abnormal connections in the brain's DMN and established a significant correlation between cingulate gyrus activity and anxiety scores, which suggests that patients with IBD are a high‐risk population for psychiatric symptoms. Brain structural changes in IBD have primarily focused on alterations in gray matter volume (Dolapcioglu & Dolapcioglu, 2015). For instance, patients with IBD may experience a decrease in the volume of the hippocampus and amygdala, while the thalamus and temporal lobe may exhibit an increase in volume (Meng et al., 2022), which regulates and incorporates emotional, cognitive, and stress responses (Goodyear et al., 2022). Agostini et al. identified a reduction in gray matter volume in the dorsolateral prefrontal cortex and anterior cingulate gyrus of patients with CD (Agostini et al., 2013). Furthermore, the authors proposed that the decline in gray matter volume in the prefrontal and marginal regions could be the underlying structural changes that contribute to the development of cognitive and emotional disorders in patients with IBD. There is a negative correlation between CD severity and the ratio of GABA+ to total creatine (GABA+/tCr), implying that a decrease in GABA+/tCr may play a role in the development of CD (Lv et al., 2018). It was also found that GABA+/tCr in the bilateral anterior cingulate gyrus increased and that Glu/tCr was positively correlated with pain scores.

In the current study, we investigated the GABA+ and Glx alterations in mPFC and its relationship with functional connectivity, as well as its association with brain structural changes in patients with IBD. We selected the mPFC as the seed region of interest (ROI) to examine alterations in its structural, functional, and metabolic characteristics. We hypothesize that: (1) there are significant differences in the structure and function of the brain between groups; (2) alterations in the structure and function of IBD are associated with depressive symptoms, which are regulated by GABA+ and Glx concentrations (Moulton et al., 2019).

## 2 METHODS AND MATERIALS

### 2.1 Participants

Forty‐two patients with IBD and 45 age‐matched healthy controls (HCs) were recruited from Lanzhou University Second Hospital. All participants were enrolled between January 2021 and July 2022 and were right‐handed. Patients with IBD were diagnosed by experienced gastroenterologists, and a reliable diagnosis was made based on patients’ clinical manifestations, laboratory tests, endoscopy, small bowel magnetic resonance imaging, and a histopathological examination. Patients were required to have maintained disease activity and be free of corticosteroids for at least 6 weeks. Individuals were excluded if any of the following conditions were met: Magnetic resonance imaging (MRI) contraindications; a history of IBD‐related abdominal surgery; a history of head trauma or neurological disease; or previous psychotropic drug use. Women underwent relevant evaluations and MRI examinations during non‐menstrual periods. The Montreal Cognitive Assessment (MoCA) scale was used to assess the participant's cognitive status. The Hamilton Depression Scale (HAMD), the Hamilton Anxiety Scale (HAMA), and the Pittsburgh Sleep Quality Inventory (PSQI) were used to collect additional psychometric data (Buysse et al., 1989). This study was approved by the Ethics Committee of Lanzhou University Second Hospital. Written informed consent was obtained from all subjects.

### 2.2 Image acquisition

All MR scans were performed on a 3.0 T MR scanner (Philips Ingenia CX, Netherlands) with a 32‐channel phased‐array head coil. During data collection, participants were asked to close their eyes and lie quietly in the scanner. We obtained whole‐brain T1‐weighted (T1w) images with a magnetization‐prepared rapid acquisition gradient echo (MPRAGE) protocol (repetition time [TR] = 7.9 ms; echo time [TE] = 3.5 ms; flip angle = 8°, matrix = 256 × 256 × 360, and voxel size = 1 × 1 × 1 mm). J‐edited MEGA‐PRESS sequence was acquired from a voxel placement (30 × 30 × 20 mm^3^) defined by orthogonal planar reconstructions (axial, sagittal, and coronal) in the center of the mPFC. The following are the MEGA‐PRESS spectral protocol parameters: TR = 2000 ms, TE = 68 ms, and 1024 acquisition points. MEGA‐PRESS editing pulses with an ON‐spectrum center of 1.89 ppm, and an OFF‐spectrum center of 7.46 ppm was averaged 100 times each, respectively. Water suppression was achieved through the method of variable power and optimized relaxation delays (VAPOR) (Tkáč & Gruetter, 2005). We also obtained an unsuppressed water spectrum with eight averages derived from identical spatial position. In addition, the Human Brain Atlas (Mai, 2015) was compared to ensure that the ROI is correctly located within the mPFC. The total scanning time for MRS was 10 min 12 s. A T2* weighted gradient echo planar imaging protocol was used: TR = 1000 ms, TE = 30 ms, field‐of‐view (FOV) = 192 × 192 mm, matrix = 64 × 64, 48 axial slices, flip angle = 84.8°, and voxel size = 3 × 3 × 3 mm^3^. Each fMRI dataset included 240 time points, that is, about 4 min 5 s.

### 2.3 Surface‐based morphometry analysis

Surface‐based morphometric analysis (SBM) was performed using the Computational Anatomy Toolbox (CAT12, http://www.neuro.uni-jena.de/cat/) on the MATLAB platform (R2016b, MathWorks, Natick, MA). First, we converted images from Dicom to the Nifti format using the DCM2Nii toolkit (https://www.nitrc.org/projects/dcm2nii/). T1w images were segmented into gray matter (GM), white matter (WM), and cerebrospinal fluid (CSF). The analysis used default parameters for segmentation, topological correction, spherical mapping, spherical registration, and cortical thickness (CT) estimation. The CT images were smoothed using a 15 mm full‐width at half‐maximum (FWHM) Gaussian kernel, as advised. We visually inspected the surface data for any artifacts and homogeneity and also conducted a statistical quality control check to ensure overall image quality. The total intracranial volume (TIV) was estimated and saved.

### 2.4 MRS analysis

The Gannet toolbox (version 3.3.0, https://markmikkelsen.github.io/Gannet-docs/) was used to evaluate the MRS data (Edden et al., 2014). The raw Philip data were preprocessed as follows: phased‐array channel combining, outlier rejection, zero padding, exponential line broadening, and subtracting the “on” and “off” spectra to get the edited difference spectrum. The concentration of the GABA+ and Glx signals (institutional units, i.u.) at 3 ppm (parts per million) was then measured relative to water using a Gaussian model. Then, the tissue within the sampling location was segmented into CSF, GM, and WM based on T1w images (Ashburner & Friston, 2005). Tissue correction for GABA‐edited MRS was performed to ensure the precision and accuracy of GABA+ and Glx quantifications (Harris et al., 2015). According to the brain clinical MRS consensus (Wilson et al., 2019), the line‐width, signal‐to‐noise ratio, and variance of Glx and GABA+ peak areas (fit‐error) were used for quality control. The line‐width of all spectra after automated shimming was <10 Hz and the fit‐error was <15%. In addition, the ratio of Glx to GABA+ served as a proxy for E/I.

### 2.5 fMRI preprocessing

The Functional Connectivity Toolbox (CONN; version 19.c, http://www.nitric.org/projects/conn) was used to analyze resting‐state fMRI data. In accordance with prior papers (Whitfield‐Gabrieli & Nieto‐Castanon, 2012), our fMRI data preprocessing went through the following steps: excluding the first 10 volumes; slice timing; realignment; indirect normalization to the Montreal Neurological Institute (MNI) template; and smoothing with a 6‐mm Gaussian kernel. The artifact removal tool (ART) was used to detect artifacts, which were subsequently incorporated as possible confounding factors in a linear regression model. Following linear detrending, the image was bandpass filtered to a frequency range of 0.01–0.08 Hz and subjected to motion regression to reduce the effects of motion and noise sources. WM, CSF, and physiological noise sources were considered to be confounding factors, and we used the “CompCor” strategy built into CONN to regress out the WM and CSF noise components (Behzadi et al., 2007). In this study, subjects with an average frame‐wise displacement (FD) > 0.2 mm were excluded (DeSerisy et al., 2020). The analyses of SBM and MRS were not relevant to these aberrant patients.

### 2.6 ROI‐to‐voxel analysis

The correlation between the average time series of selected seed points and all remaining voxel time series in the whole brain was evaluated by seed‐based connectivity analysis in the CONN toolbox using GLM (no weighting was applied) (Whitfield‐Gabrieli & Nieto‐Castanon, 2012). The predefined seeds were placed within the customized mPFC cubes. We employed the Advanced Normalization Tools (ANTs, http://stnava.github.io/ANTs/) to co‐register the binary mask produced from ^1^H‐MRS data into MNI standard space (Avants et al., 2014). In practical terms, the binary mask was transformed to MNI standard space using the parameters after inverse transformation using the conversion matrix configured from individual T1 to MNI standard space (Aranovich et al., 2016). The resolution of the seed points used was consistent with the size of the fMRI (3 × 3 × 3 mm^3^).

### 2.7 Statistical analyses

Statistical analyses were conducted using the open‐source statistical computer program R (https://www.r-project.org, version 4.2.1). Independent sample *t*‐tests were used to evaluate differences in demographics and clinical measures between patients with IBD and controls. The Shapiro–Wilk test was used to determine whether the data corresponded to a normal distribution. Differences in the gender of the two groups were analyzed using the chi‐square test. All statistical analyses of neuroimaging data were performed using a general linear model (GLM) between the two groups after adjusting for age, gender, and years of education. For cortical thickness, the cluster‐level family‐wise error rate (FWE) was assessed for multiple comparisons with an extended threshold of 100 vertices after an initial cluster‐forming threshold of *p* < 0.05. For functional connectivity, an FWE‐corrected threshold of *p* < 0.001 was applied to identify statistically significant clusters. Then, the mean cortical thickness and functional connectivity values of the discriminative clusters were extracted. Partial Pearson's correlation coefficients controlling for gender were calculated. At last, we used mediation analysis to test whether the GABA+ or Glx mediated the effects of functional connectivity or CT on the HAMD score using the “mediation” package with 5000 permutation tests in R (Computing, 2013). All analyses, including mediation, were corrected for multiple comparisons at *p* < 0.05 using a Bonferroni correction.

## 3 RESULTS

### 3.1 Demographic and clinical characteristics

The data of 18 participants were excluded from the analysis due to excessive head motion, aberrant anatomical scans, or poor MRS quality control. Hence, data from 37 patients and 32 HCs were included in the final analysis. Compared with the HCs, patients with IBD had greater symptoms of anxiety, depression, and sleep disorders (all *p* < 0.001, Table 1).

**TABLE 1:** Demographic characteristics and neuropsychiatric data of participants.

	HC (*N* = 32)	IBD (*N* = 37)	*p* value	Statistic value
Age (*y*)	43.3 (11.9)	45.8 (12.8)	0.408	−0.833
Gender (male %)	16 (50.0)	9 (45.0)	0.938	0.067
TIV (cm^3^)	1470 (134)	1460 (181)	0.710	0.374
HAMD	4.50 [2.25, 6.00]	16 [7.5, 19.5]	<0.001	−5.89^a^
IBDQ	NA	150.85 (42.29)	–	–
PSQI	3.75 (1.85)	6.75 (2.97)	<0.001	0.754
VAS	NA	4.55 (2.09)	–	–
HAMA	2.88 (1.79)	13.40 (3.41)	<0.001	2.302
MoCA	26.95 (1.52)	27.18 (2.39)	0.898	0.077
SF‐MPQ	NA	13.70 (5.11)	–	–
Disease duration (*y*)	NA	6.00 [3.00, 8.00]	–	–
VSI	67.50 [60.00, 89.00]	58.00 [49.75, 60.25]	<0.001	0.798^a^
PCS	1.66 (3.41)	12.65 (11.07)	<0.001	1.037
SF‐36	86.50 [79.75, 92.00]	69.50 [63.75, 76.25]	<0.001	1.035^a^
GSRS	NA	8.00 [6.50, 11.00]	–	–^a^
BMI	19.8 [18.02, 22.41]	21.45 [19.56, 22.86]	0.03	−2.450^a^
Medication (*n*)				
Conventional immunosuppressants	NA	19	–	–
Anti‐TNF antibody	NA	26	–	–
Pain medication	NA	5	–	–

*Note*: Data are presented as mean ± SD or median [IQR] or N (%).

Abbreviations: BMI, body mass index; GSRS, Gastrointestinal Symptom Rating Scale; HAMA, Hamilton Anxiety Rating Scale; HAMD, Hamilton Depression Rating Scale; IBDQ, Inflammatory Bowel Disease Questionnaire; MoCA, Montreal Cognitive Assessment; NA, not available; PCS, Peritraumatic Distress Inventory; PSQI, Pittsburgh Sleep Quality Index; SF‐36, Short Form‐36 Health Survey; SF‐MPQ, Short‐Form McGill Pain Questionnaire; TIV, Total Intracranial Volume; TNF, tumor necrosis factor; VAS, Visual Analog Scale; VSI, Visceral Sensitivity Index; y, year.

^a^ Data does not follow a normal distribution, and the Mann–Whitney *U* test was used for statistics.

### 3.2 Cortical neurotransmitter group differences (MRS)

**FIGURE 1:**
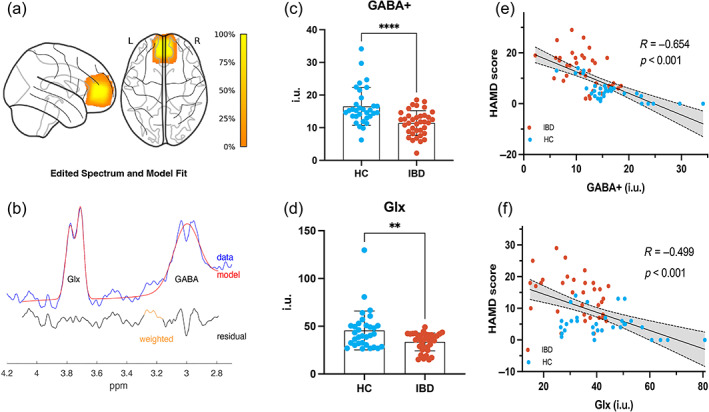
^1^H‐MRS results for the medial prefrontal cortex (mPFC). (a) The voxel size and location and the overlap in MRS voxels for all participants in both groups. (b) The edited γ‐aminobutyric acid (GABA) and combined resonance of glutamate and glutamine (Glx) peaks obtained using Magnetization‐Encoded Gradient Adapted Spectroscopy PRESS (MEGA‐PRESS) fitted by the Gannet toolbox. The edited spectrum (black line) and fitted curve (red line). (c, d) Histogram representing case–control differences of GABA+ and Glx, respectively. (d, f) Scatter plots showing the correlation between GABA+ or Glx and the HAMD score. Light blue indicates HC subjects and orange indicates patients with IBD.

Throughout the scan, we checked for excessive motion, which was validated by spectral quality checks. The FWHM, SNR, and fraction of the tissue are presented in Table S1. The mean and standard deviation of GM/WM/CSF were 58.8 ± 6.6%, 22.4 ± 6.9%, and 18.8 ± 7.1%, respectively. Figure 1a shows the typical GABA+ and Glx spectral curves after fitting with the Gannet toolkit. Figure 1b shows the fitted GABA+ and Glx peaks obtained from MEGA‐PRESS. Compared with HCs, patients with IBD had substantially lower GABA+ and Glx concentrations in the mPFC region (GABA: IBD = 11.43 ± 3.79, HC = 16.51 ± 5.78, *p* < 0.001, Figure 1c; Glx IBD = 33.60 ± 9.39, HC = 45.53 ± 20.38, *p* = 0.0074, Figure 1d). The GABA+ level was negatively correlated with the HAMD score across all participants (*r* = −0.654, *p* < 0.001, Figure 1e). The Glx level was negatively correlated with the HAMD score (*r* = −0.499, *p* < 0.001, Figure 1f). The results of the two‐sample *t*‐test showed that there was no statistically significant difference in the E/I ratio between the two groups (mean ± SD: IBD = 3.13 ± 1.13, HC = 2.87 ± 1.15, *t* = 0.95, *p* = 0.35).

### 3.3 Cortical group differences (SBM)

**FIGURE 2:**
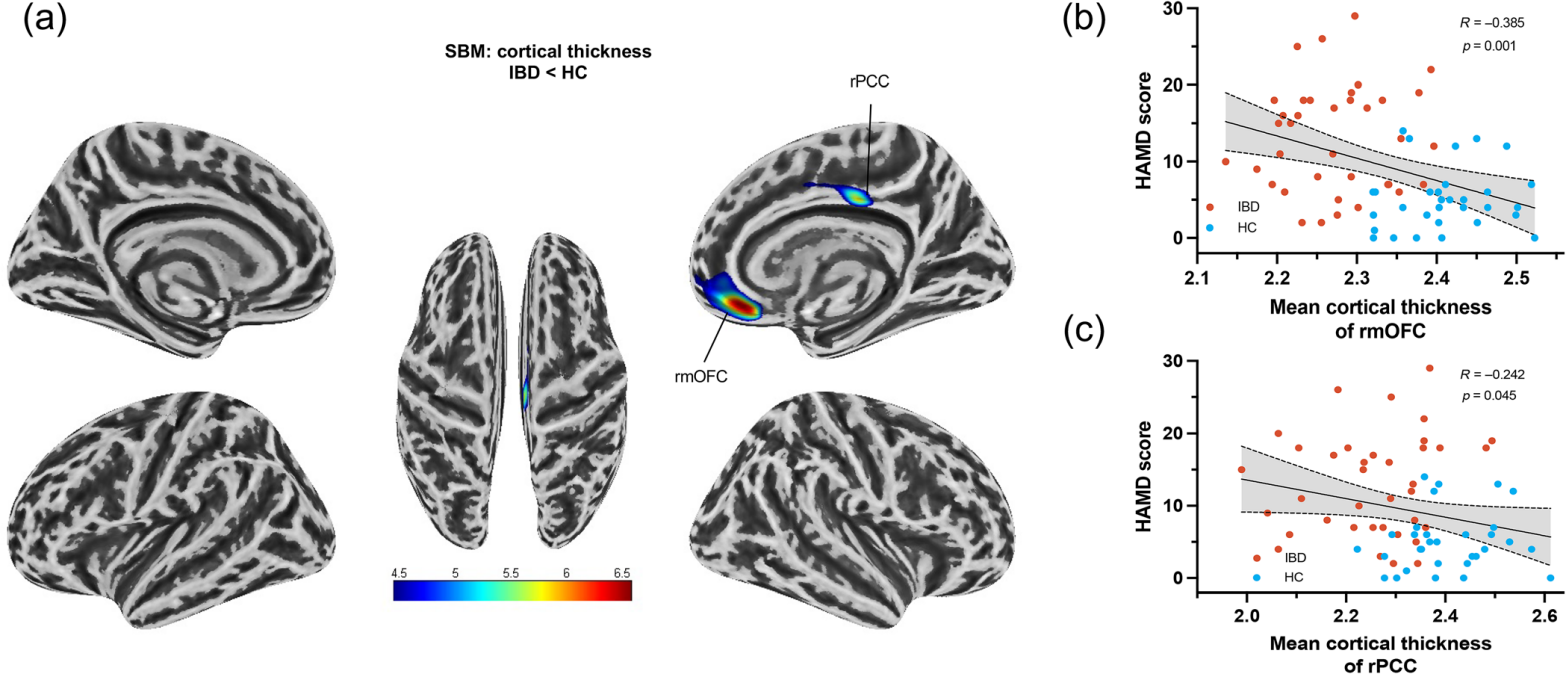
Vertex‐wise comparisons of cortical thickness between the IBD group and the healthy control group and correlation analysis between the cortical thickness of discriminative clusters and HAMD scores. (a) A cluster‐level FWE‐corrected *p* < 0.05 threshold was used to threshold statistical maps. (b, c) Mean adjusted cluster cortical thickness of correlations to HAMD scores are shown with scatter plots. *rmOFC*: right medial orbitofrontal cortex, *rPCC*: right posterior cingulate cortex. Light blue indicates the healthy control group and orange indicates the IBD group.

SBM analysis revealed that CT decreased in two clusters (Figure 2a and Table 2), consisting of the right medial orbitofrontal cortex (rmOFC) and right posterior cingulate cortex (rPCC). In addition, we extracted the mean CT values from clusters with significant differences for correlation analysis. The results indicated that there was a negative correlation between the HAMD score and the mean CT in the rmOFC (*r* = −0.385, *p* = 0.001, Figure 2b) as well as the mean thickness of the cortical ribbon in the rPCC (*r* = −0.242, *p* = 0.045, Figure 2c).

**TABLE 2:** Differences in SBM and seed‐based functional connectivity.

Method	Brain region	Abbreviation	Contrast	T value	MNI			Vertex/voxel size
					X	Y	Z	
SBM	Right medial orbitofrontal cortex	rmOFC	IBD < HC	6.6	6	33	−14	1364
	Right posterior cingulate cortex	rPCC	IBD < HC	5.8	3	−20	35	564
Functional connectivity	Left precuneus cortex	PC	IBD < HC	4.63	−6	−69	24	68
	Left frontal pole	FP	IBD > HC	−4.86	−39	48	15	43
	Posterior cingulate cortex	PCC	IBD < HC	5.17	−6	−48	6	36

*Note*: All results are corrected at a cluster‐level FWE *p* < 0.05 threshold.

Abbreviations: MNI, Montreal Institute of Neuroimaging; SBM, surface‐based morphometry.

### 3.4 Seed‐based connectivity results

**FIGURE 3:**
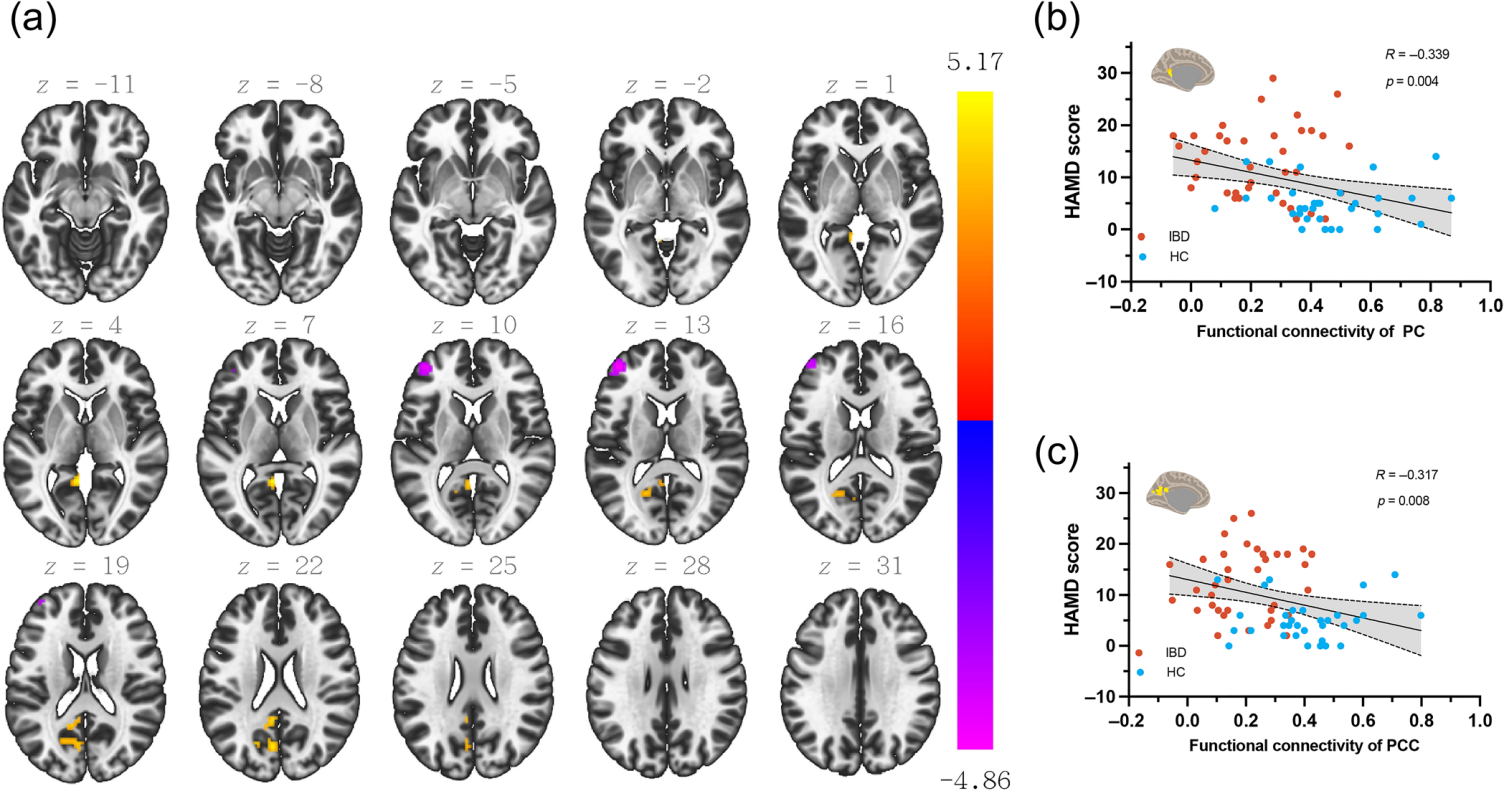
Results of seed‐based functional connectivity between the mPFC and all other voxels in the whole brain, and correlation analyses between the functional connectivity of discriminative clusters and HAMD scores. (a) Higher functional connectivity was detected within the left precuneus cortex and left posterior cingulate cortex (PCC). Decreased functional connectivity was measured within the left frontal pole (FP). FEW‐corrected with a threshold of *p* < 0.05. (b, c) Mean functional connectivity correlations with HAMD scores are shown with scatter plots. Light blue indicates the healthy controls group, and orange indicates the IBD group.

Based on the results of seed‐based connectivity, using the MRS mask as the source seed, there were three brain regions in the significantly related cluster. The analyses revealed the presence of three distinct related brain regions, namely the left precuneus cortex (PC), left frontal pole (FP), and posterior cingulate cortex (PCC) (refer to Figure 3 and Table 2). A significant negative correlation was observed between HAMD scores and the functional connectivity of both the mPFC‐PC region (*r* = −0.339, *p* = 0.004, Figure 3b) and the mPFC‐PCC region (*r* = −0.371, *p* = 0.008, Figure 3c).

### 3.5 Mediation analysis and correlation results

**FIGURE 4:**
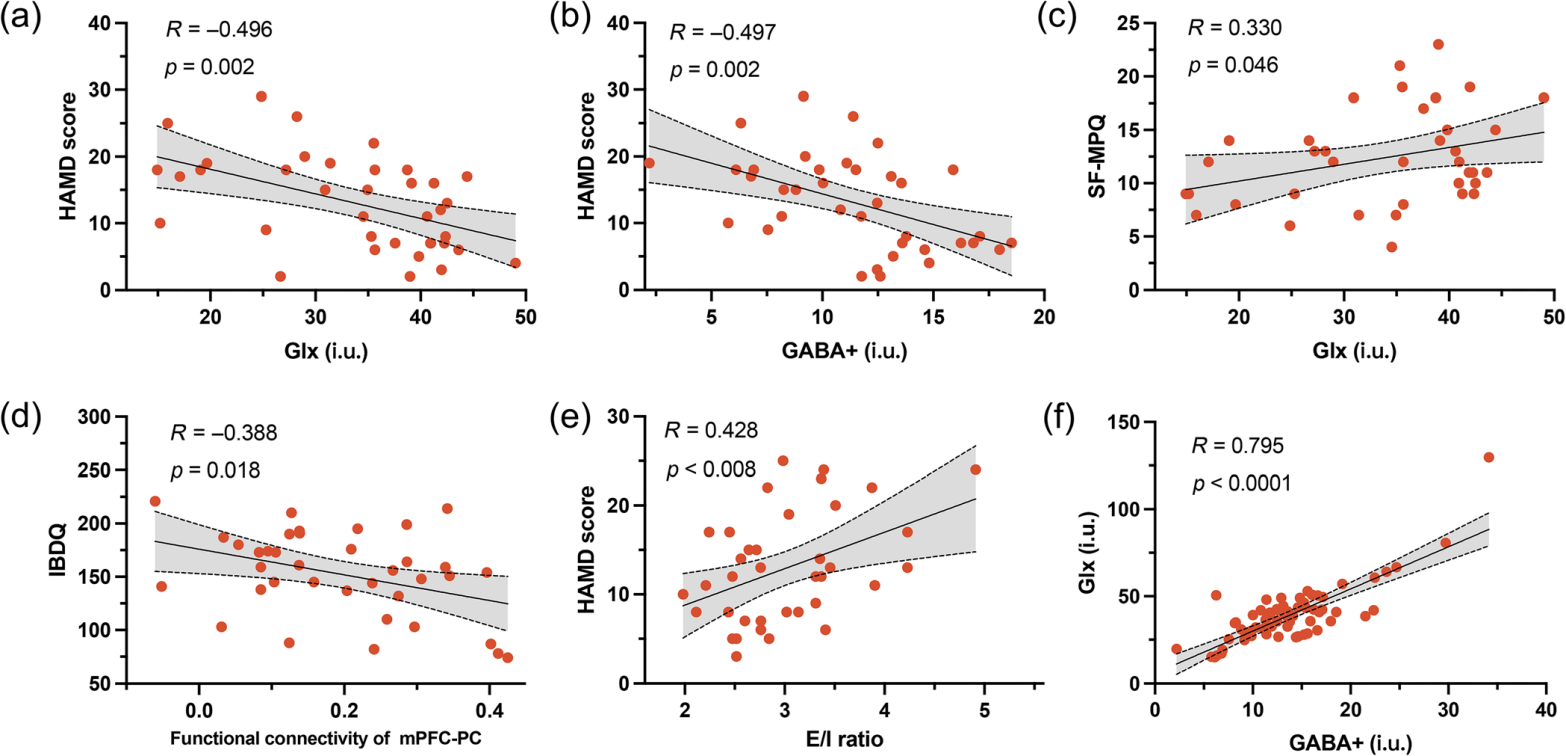
Correlations between neuroimaging and clinical data in the IBD group. (a, b) Partial correlation was used to detect the relationship between GABA+ and Glx concentrations and the HAMD score, controlling for age. (c) Partial correlation results between Glx and Short‐Form McGill Pain Questionnaire (SF‐MPQ) score, controlling for age (the SF‐MPQ reflects the severity of pain). (d) Functional connectivity between the mPFC and left frontal pole (FP) had a negative correlation with scores on the Inflammatory Bowel Disease Questionnaire (IBDQ). (e) The Glx/GABA+ ratio had a positive correlation with the HAMD score. (f) GABA+ had a positive correlation with Glx level.

The study's results showed a significant negative correlation between the HAMD score and both GABA+ and Glx concentrations in patients with IBD, as illustrated in Figure 4a and Figure 4b. A positive correlation was observed between Glx concentration and the Short‐Form McGill Pain Questionnaire score (*R* = 0.330, *p* = 0.046, Figure 4c) in patients with IBD, while a negative correlation was found between the functional connectivity of the mPFC–PC and the IBD questionnaire score (*r* = −0.388, *p* = 0.018, Figure 4d). The results of the correlation analysis indicated a significant positive correlation between the ratio of Glx to GABA+ and the HAMD scores of the IBD group (*r* = 0.428, *p* = 0.008, Figure 4e). There was a significant positive correlation between GABA+ and Glx concentrations (*r* = 0.795, *p* < 0.001, Figure 4f). The gray matter fraction in each voxel was not correlated with the Glx or GABA measurements (*p* > 0.25). No significant diagnosis × sex interactions were discovered.

**FIGURE 5:**
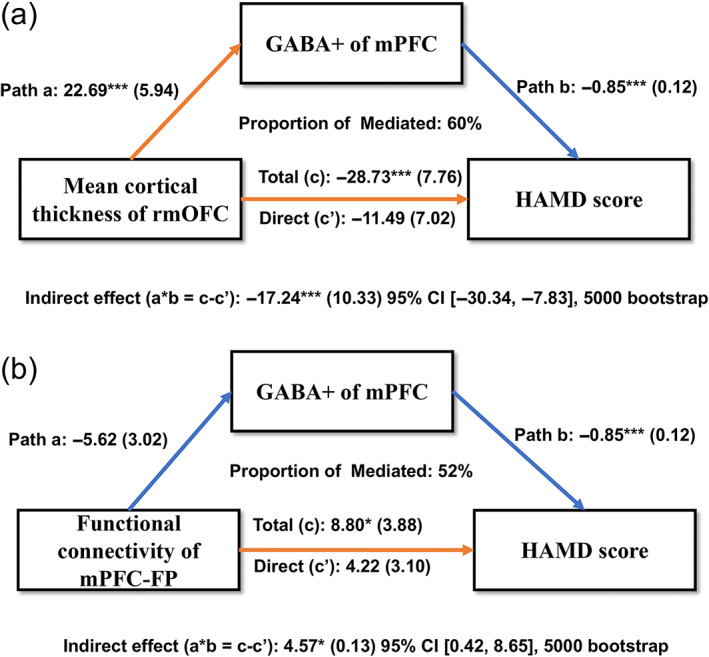
Mediation results of the effects of GABA+ on the association between brain structure/function changes and the HAMD score. In (a), cortical thickness of the rmOFC was entered as a predictor; in (b), functional connectivity between the mPFC and FP was entered as a predictor. The results show that GABA+ mediated the effects of gray matter loss on HAMD, and it also mediated the effects of functional connectivity on HAMD. The paths (path a, b, and c′) and mediation effects (path a × b) are labeled with path coefficients, and their standard errors are shown in parentheses. Orange represents a positive regulatory effect, and blue represents a negative regulatory effect. *p < 0.05, ***p < 0.001.

The results presented in Figure 5a show there was a relationship between the concentration of the neurotransmitter GABA+ and depression score (*r* = 0.422, *p* = 0.0003). Specifically, a decrease in GABA+ was related to an increase in the depression score (path b). Moreover, changes in CT affected GABA+ concentrations (path a). GABA+ significantly explained 60% of the overall effect of the link between the independent variable rmOFC and the dependent variable HAMD score, showing that GABA+ plays a considerable role in mediating this relationship. Furthermore, GABA+ also mediated the effect of the functional connection between FP and depression symptoms (Figure 5b). No similar mediating effect was found for the Glx.

## 4 DISCUSSION

In this study, we used multimodal MRI analysis methods to probe brain abnormal functional connectivity, cortical thickness, and its association with mPFC GABA+ and Glx concentrations in patients with IBD. We demonstrated altered brain functional connectivity of the mPFC with the left PC, the PCC, and the left frontal pole. The cortical thickness was decreased in two clusters that included the right medial orbitofrontal cortex and the right posterior cingulate cortex. Moreover, the GABA+ and Glx exhibit a decrease in mPFC and the GABA+ have a significant mediating effect on the association between changes in regulatory functional connectivity and depressive score. The same effect also occurs between reduced cortical thickness and depressive symptoms. These data and specifically, observed neurotransmitter changes in mPFC, provide new insights into the pathology of depression in patients with IBD.

IBD is a systemic disease that involves an imbalance in the internal environment's homeostasis (Dave et al., 2014). Abnormal structural changes in the brains of patients with IBD may be associated with several factors, such as pain, inflammation, stress, and emotions (Oligschlaeger et al., 2019). Inflammation of the gut or transmission of harmful stimuli travels along the spinal and vagus nerves, relayed to the brain through afferent sensory fibers from the gastrointestinal tract (Browning et al., 2017). This can lead to changes in cortical or subcortical plasticity in the brain (Craig et al., 2022). Inflammatory signals from the gut can induce apoptosis of astrocytes and oligodendrocytes, as well as activate microglia, macrophages, and endothelial cells in the brain to alter the excitability of the central nervous system (Olude et al., 2022). Long‐term chronic stress has a negative effect on the PFC (Pizzagalli & Roberts, 2022). According to brain imaging studies (Wu et al., 2023), the PFC exhibits a selective volumetric reduction in several neuropsychiatric disorders related to stress, especially major depressive disorder (MDD). A meta‐analysis of depression also showed that gray matter volume in the frontal lobe region decreased the most (Wise, Radua, et al., 2017). Neurobiological alterations within the mPFC and its adjacent brain regions distinctly contribute to the manifestation of depressive symptoms (Guo et al., 2012). The medial OFC and mPFC are two adjacent and closely related brain regions. They are both located on the medial side of the prefrontal cortex, and they have some functional overlap. Studies have shown that the mPFC can modulate medial OFC responses to rewards and punishments and decision‐making (Euston et al., 2012). At the same time, the medial OFC may also participate in the regulation of emotion and social cognition by sending information to the mPFC (Delaparte et al., 2019; Kennerley & Walton, 2011). In depression‐related diseases, there is more inflammation in the medial OFC compared with healthy people (Yu et al., 2022). Therefore, the significant decrease in cortical thickness in rmOFC found in our study may be due to the local response of the brain to systemic inflammation. Investigating the reasons for the presence of low‐level inflammation in the brain during illness development may explain the high comorbidity rate between depression and IBD (Craig et al., 2022).

Individuals with IBD exhibit structural and functional alterations in the DMN (Thomann et al., 2021). The PCC is located in the posterior medial region of the parietal lobe and, together with the mPFC, precuneus, and hippocampus, are key nodes of the DMN (Fransson & Marrelec, 2008; Kraynak et al., 2018). The input of the PCC may be biased toward unhappy or sad events and increased functional connectivity between it and the lateral orbitofrontal cortex has been observed in patients with depression (Cheng et al., 2018). Wise et al. found that greater connectivity variability was detected in the major depression between mPFC and PCC (Wise, Marwood, et al., 2017). Besides, we also found hypoconnectivity in the left PC and hyperconnectivity in the left FP with the mPFC. There is a statistical correlation between hypoconnectivity in the left PC and IBDQ, indicating that functional connectivity may reflect the patient's health‐related quality of life (Nawani et al., 2019). While the association between GABA+ and functional connectivity was only detected in the left FP, this is consistent with growing evidence of alterations in pain‐related brain networks, indicating that chronic pain affects functional synergy in the DMN (Kucyi et al., 2014). The functional connectivity of mPFC‐FP has a negative correlation with the decrease in GABA+ concentration in the mPFC, which indicates that the decrease in inhibition drives the de‐correlation of neural activity (Chini et al., 2022). According to research (Dixon et al., 2017), strengthening the relationship between the mPFC and the FP may be beneficial to mental wellness. Our findings might indicate that the brain is compensating in this manner.

MRS can answer functionally relevant questions in clinical neuroscience (Bednarska et al., 2019). GABA+ regulates neuronal excitability through its primary inhibitory action in the CNS. The reduction of GABA+ concentrations implies the loss of direct inhibition (Romaus‐Sanjurjo et al., 2018). The loss of GABA+ intermediate neurons is predominantly brought on by cytotoxins, such as glutamate, inflammatory cytokines, and reactive oxygen compounds (Haroon et al., 2017). Low GABA+ concentrations may indicate a decrease in the density and size of GABAergic intermediate neurons, which could be reflected by decreased cortical thickness. Glial cell activation in chronic pain may result in neurotransmitter abnormalities in a variety of ways (Naylor et al., 2019). Chronic stress from IBD could decrease the function of GABA‐A receptors, mainly by reducing neuroactive steroid synthesis (Duman et al., 2019). Prefrontal GABAergic neurotransmission appears to be downregulated by psychological stress (Rajkowska et al., 2007). Recent studies suggest that GABA is also crucial for improving immune and inflammatory responses (Tian & Kaufman, 2023). A decrease in GABA+ concentration was discovered in IBD, which may indicate a passive reduction in GABA+ in immune and inflammatory responses (Hernández‐Alonso et al., 2020). Separately, the results herein showed that GABA+ concentration was negatively correlated with the HAMD score, indicating that reduced GABA+ concentration may be involved in the pathogenesis of IBD‐concomitant depressive symptoms. Mediation analysis revealed that changes in GABA+ concentration in the mPFC modulated the relationship between brain structural and functional changes and depressive symptoms in patients with IBD. We did not find a direct correlation between GABA+ and Glx concentrations and functional connectivity (all *p* > 0.05). However, throughout the entire sample, it could be seen that the cortical thickness of the rmOFC was positively correlated with a reduction in GABA+ concentration. GABA+ may be a regulating factor in the compensatory mechanism of increased functional connectivity in the prefrontal lobe (Delli Pizzi et al., 2017). We believe that the functional and structural changes mediated by neurotransmitter depletion in the mPFC may be a key mechanism underlying the development and maintenance of chronic pain, comorbid mood, and emotional disorders (O'Neill et al., 2016).

The Glx concentration mainly evaluates the glutamate and glutamine concentrations throughout the entire intracellular pool in the spectroscopic voxels (Hasler et al., 2007). Previous studies using MRS have furnished proof that brain responses are modulated by emotional involvement and that alterations in the glutamate system are linked to anxiety (Mayer et al., 2015). Compared with the HCs, IBD patients showed a significant decrease in Glx in the mPFC and a negative correlation with the depression score, which is consistent with our hypothesis, indicating the presence of glutamate dysfunction. Our study also found a positive correlation between decreased Glx concentration and pain scores, which may indicate that the Glx reflects pain sensitivity in the brain (Archibald et al., 2020). This has also been shown in the spinal cord of rats, which indicates that glutamate may play a role in the emergence of chronic pain (Salvemini et al., 2011; Yang & Chang, 2019). There is controversy surrounding the secretion of Glx in the brains of patients with chronic pain (Terumitsu et al., 2022). Some studies have reported insignificant case–control differences (Brennan et al., 2017; Godlewska et al., 2015). In addition, the results for Glx have not been consistent. Some studies have reported higher Glx concentration in patients with MDD (Kantrowitz et al., 2021), while others have reported lower Glx concentration compared with the healthy control group (Moriguchi et al., 2019). A previous study of metabolite changes in the bilateral anterior cingulate cortex (ACC) of patients with painful CD with abdominal pain found that the ratio of Glx to tCr was higher in the painful CD group than in the non‐painful CD group (Vogt, 2013), which is contrary to our results and possibly due to the different distribution of Glx concentrations between different brain regions, or due to inconsistent reference materials (Zhou & Danbolt, 2014). Although our research suggests that there is no statistical correlation between the decrease in Glx concentrations and cortical thickness, imbalances in glutamate signaling are implicated as a potential cause of the observed structural changes in these regions (Duncan et al., 2013). This may be the reason why our sample size is relatively small. The two major neurotransmitter pools (GABA+ and Glx) in the mPFC area were found to be positively correlated. Our results seem to support the positive link between the release and metabolism of glutamate and GABA+ that has been observed in neurophysiological research (Magistretti et al., 1999).

To our knowledge, this is the first study to investigate emotional disorders in patients with IBD using the ratio of Glx to GABA+ (proxy of E/I). A common characteristic found in models of normal brain function is the existence of an equilibrium between the levels of excitatory and inhibitory activity, which is referred to as the E/I balance (Vogels & Abbott, 2009). Theoretically, a decrease in GABA concentration may result in the suppression of glutamic activity (Levy & Degnan, 2013). There is still no consensus on the changes in brain neurotransmitters that are related to depression (Moncrieff et al., 2022). Most research reports indicate a decrease in GABA concentration in patients with depression compared with a healthy control group (Romeo et al., 2018; Sanacora et al., 2004). To circumvent contradictory findings between Glx and GABA concentrations, a ratio method was employed in our study. The findings suggest that there are no significant group differences in the Glx/GABA+ ratio of individuals with IBD, but the E/I is strongly associated with depression severity in patients with IBD. This implies that changes in neurotransmitter levels on a local level may be related to depression symptoms in individuals with IBD. The release of neurotransmitters in the brain is modulated and controlled by a variety of internal and external factors, such as the gut microbiota, neuronal activity, the hypothalamus‐hypophysis‐adrenal axis, and diet (Chen et al., 2021). It can be inferred from previous research that patients with IBD have an imbalance in their gut microbiota (Alam et al., 2020), which may affect the levels of neurotransmitters in the brain. We believe that depression is caused by the impairment of neuroplasticity, which may be caused by the imbalance of neurotransmitters (Liang et al., 2018).

The limitations of the current study include: (1) We were unable to investigate the causality existing between IBD and depression symptoms because this study was cross‐sectional in nature; (2) no subgroup analyses were conducted because the sample size for our final data analysis was relatively small (to ensure the reproducibility of the research, larger samples will need to be used in future studies); and (3) our study did not contain any pertinent inflammatory signs in the correlation analyses. Systemic low‐grade neuroinflammation brought on by IBD is one of the possible reasons for anxiety and depression disorders among patients with IBD (Craig et al., 2022). Future research on the mechanisms of IBD should incorporate different inflammatory indicators to explore the relationship between immunology, neuroinflammation, and the onset of IBD neuropathy; (4) Our study exclusively focused on examining metabolic information within the mPFC, limiting the generalizability of our findings to other cortical brain regions; (5) Due to practical and ethical issues, as patients are in an active phase, we cannot rule out the potential confounding effects of drugs.

## 5 CONCLUSION

Our study provides detailed evidence for the neurochemical basis of depressive symptoms in patients with IBD. Patients with IBD presented with significantly lower GABA+ and Glx in the mPFC, compared with healthy controls. The decrease in GABA+ concentrations may mediate the relationship between structural and functional alterations in the brain and depression scores. This may represent an important indicator of brain changes in IBD. It has been found that GABA+ plays a role in the relationship between the gut microbiota and the central nervous system (Carabotti et al., 2015). In the future, the study of intestinal microbiology and the metabolome will help us understand the potential interactions with the brain in patients with IBD.

## AUTHOR CONTRIBUTIONS

Jun Wang and Kun Xu conceptualized the study. Guangyao Liu and Jun Wang conducted the study, analyzed the data, wrote the manuscript, and approved the final version to be published. Jing Zhang, Wenjing Huang, and Kai Ai critically revised the manuscript and approved the final version.

## FUNDING INFORMATION

This study was supported by the National Natural Science Foundation of China, Grant/Award Number: 81960309, 82160326; the Gansu Province Clinical Research Center for Functional and Molecular Imaging, Grant/Award Number: 21JR7RA438; the National Natural Science Foundation of China, Key R&D Plan of Gansu Province, Grand/Award Number: 22YF7FA089; the Scientific Research Project of Gansu Health Industry, Grant/Award Number: GSWSKY2021‐031.

## CONFLICT OF INTEREST STATEMENT

There are no declared conflicts of interest.

## Supporting information


**Table S1.** Individual fit quality metrics for quantification of GABA+/Glx in mPFC. Group differences in each of the fit quality metrics are given at the bottom of the table.

## Data Availability

The data that support the findings of this study are available on request from the corresponding author. The data are not publicly available due to privacy or ethical restrictions.
