# Supplementary material for: The role of neurotransmitters in mediating the relationship between brain alterations and depressive symptoms in patients with inflammatory bowel disease
Source: Hum Brain Mapp. 2023 Aug 2;44(16):5357–71. doi: 10.1002/hbm.26439 (PMC10543356; doi:10.1002/hbm.26439)
Supplement: Supplementary file 1 — Table S1. Individual fit quality metrics for quantification of GABA+/Glx in mPFC. Group differences in each of the fit quality metrics are given at the bottom of the table. [file HBM-44-5357-s001.docx]

**Table S1.** Individual fit quality metrics for quantification of GABA+/Glx in mPFC. Group differences in each of the fit quality metrics are given at bottom of table.

| **Group** | **GABA+** | | | **Glx** | | | **Tissue fraction** | | |
| --- | --- | --- | --- | --- | --- | --- | --- | --- | --- |
|  | **FE** | **FWHM** | **SNR** | **FE** | **FWHM** | **SNR** | **fGM** | **fWM** | **fCSF** |
| **HC (n = 32)** | 9.28 ± 2.36 | 21.63 ± 5.42 | 12.25 ± 3.96 | 8.96 ± 3.43 | 16.63 ± 2.09 | 12.85 ± 3.00 | 0.59 ± 0.07 | 0.21 ± 0.07 | 0.20 ± 0.09 |
| **IBD (n = 37)** | 11.68 ± 8.92 | 20.80 ± 3.19 | 11.63 ± 4.00 | 10.16 ± 8.15 | 16.37 ± 2.10 | 13.66 ± 5.48 | 0.58 ± 0.06 | 0.24 ± 0.08 | 0.18 ± 0.05 |
| **t/U** | 581* | 496* | 0.52 | 549* | 0.51 | 573* | 0.64 | 1.49 | 541.5* |
| ***P*-value** | *0.90* | *0.25* | *0.65* | 0.61 | *0.61* | *0.82* | 0.53 | *0.14* | *0.55* |

*Note:* the asterisk (*) represents that the data does not follow a normal distribution, and the Mann-Whitney U test is used to conduct statistical analysis. Data are presented as Mean ± SD; FE, fit error; FWHM, full width at half maximum; SNR, signal to noise ratio. fGM, fraction of gray matter; fWM, fraction of white matter; fCSF, fraction of cerebrospinal fluid.
